# A Protocol for Generating and Exchanging (Genome-Scale) Metabolic Resource Allocation Models

**DOI:** 10.3390/metabo7030047

**Published:** 2017-09-06

**Authors:** Alexandra-M. Reimers, Henning Lindhorst, Steffen Waldherr

**Affiliations:** 1 Department of Mathematics and Computer Science, Freie Universität Berlin, 14195 Berlin, Germany; 2 International Max Planck Research School for Computational Biology and Scientific Computing, Max Planck Institute for Molecular Genetics Berlin, 14195 Berlin, Germany; 3 Institute for Automation Engineering, Otto-von-Guericke-Universität Magdeburg, 39106 Magdeburg, Germany; 4 KU Leuven, Department of Chemical Engineering, 3001 Leuven, Belgium; steffen.waldherr@kuleuven.be

**Keywords:** constraint-based modeling, optimality, metabolic networks, SBML

## Abstract

In this article, we present a protocol for generating a complete (genome-scale) metabolic resource allocation model, as well as a proposal for how to represent such models in the systems biology markup language (SBML). Such models are used to investigate enzyme levels and achievable growth rates in large-scale metabolic networks. Although the idea of metabolic resource allocation studies has been present in the field of systems biology for some years, no guidelines for generating such a model have been published up to now. This paper presents step-by-step instructions for building a (dynamic) resource allocation model, starting with prerequisites such as a genome-scale metabolic reconstruction, through building protein and noncatalytic biomass synthesis reactions and assigning turnover rates for each reaction. In addition, we explain how one can use SBML level 3 in combination with the flux balance constraints and our resource allocation modeling annotation to represent such models.

## 1. Introduction

In recent years, the systems biology of metabolism has moved more and more from classical metabolic network study towards the study of growth as a result of an optimized cellular economy. This idea of studying growth strategies using resource allocation models has been initiated by Molenaar et al. [1] in 2009. In their article, Molenaar et al. used a small dynamic model of a self-replicating system to explain how overflow metabolism arises by means of tradeoffs between different growth strategies. Further on, Goelzer et al. [2] introduced resource balance analysis (RBA), as a means of predicting the cell composition of bacteria in a specific (constant) environment through a convex optimization problem that includes the bioenergetic cost of producing the enzymes required in a pathway. As a similar approach, Palsson and colleagues introduced the idea of an integrated model of metabolism and gene expression (ME model) as a means to explore the relationship between genotype and phenotype using biochemical representations of transcription and translation processes [3,4]. Their research group then continued with an ME model of *Escherichia coli* [5]. With the COBRAme package [6], a computational framework for building and manipulating ME models is provided. Also experimental studies focused on relating absolute protein abundances to how metabolic pathways balance production costs and activity requirements [7].

These formalisms have then been taken a step forward, towards understanding how resources are distributed in a dynamically changing environment by means of a dynamic enzyme-cost flux balance analysis (deFBA) [8] and conditional flux balance analysis (cFBA) [9]. This has then been taken to the genome scale by studying the optimal glycogen and metabolite partitioning dynamics under a day-night cycle in a cyanobacterium using a dynamic resource allocation model [10].

Such dynamic resource allocation models have a wide area of application. One such an example is the study of microorganisms growing in industry-scale bioreactors. There, the organism has to balance resources not only in order to grow optimally, but also in order to withstand transitions through local heterogeneities of the reactor. The ability to take such transitions into account within metabolism has been shown to be crucial for survival [11]. Moreover, an extension of the deFBA formalism has been developed in order to predict the optimal resource allocation in an environment where such uncertainties are present [12].

Given all these recent developments, we believe that there is a need to establish a protocol for building a metabolic resource allocation model. However, to the best of our knowledge, there exists no generic guideline that details how to proceed in the construction of large-scale metabolic resource allocation models, together with possible sources of the relevant parameters. Moreover, there exists so far no specification for defining and exchanging these models that would be similarly useful as the current SBML standard for kinetic and metabolic flux balance models.

**Figure 1:**
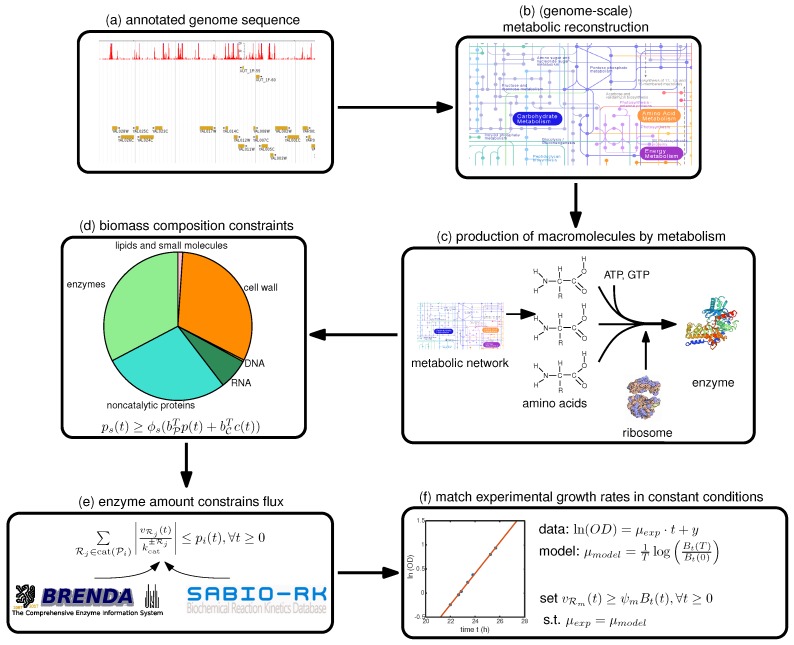
Protocol for generating a deFBA model. From an annotated genome sequence (**a**) of the organism of interest the metabolic network (**b**) is reconstructed following instructions in [15]. Given the gene-reaction mapping and the annotated genome sequence, the enzymes and ribosomes (**c**); and their synthesis reactions are added to the stoichiometric matrix (see Section 4). Next, the biomass composition constraints (**d**) should be set up using information from the biomass objective function of the metabolic network model (see Section 5). Then reaction turnover rates (**e**) sourced from literature and online databases should be added (see Section 6). Lastly but most importantly, the deFBA model should be fine tuned to match experimental growth rates (**f**) obtained in the laboratory (see Section 7). Images retrieved from: (**a**) http://goo.gl/aBNfPz [16,17]; (**b**) http://www.genome.jp/kegg-bin/show_pathway?map01100 [18]; (**c**) http://www.genome.jp/kegg-bin/show_pathway?map01100 [18], http://pdb101.rcsb.org/motm/10 [19], https://swissmodel.expasy.org/repository/uniprot/P04806 [20].

Therefore, we focus in this paper on a step-by-step guide towards constructing such a model, summarized in Figure 1, with a focus on the deFBA formalism which is described in the Methods section. Note however, that these guidelines can be used as well for building cFBA, RBA and ME models. We detail here all the necessary information as well as which databases may be used to retrieve it (Table 1). To facilitate exchange among researchers, we furthermore propose a new SBML specification, called resource allocation modeling (RAM) [13]. This specification allows encoding such models in the SBML format using the Flux Balance Constraints extension [14].

In addition to this protocol, we provide software in Python 2.7 (implementation can be found at: https://bitbucket.org/hlindhor/defba-python-package) as well as MATLAB R2016a (implementation is available at https://github.com/alexandra-m-reimers/deFBA) for reading and writing resource allocation models using our SBML specification as well as for solving deFBA problems. We would like to note that these models are strongly organism-dependent. Therefore, the modeler is still required to decide which key processes are modeled and which level of detail is used for their particular application.

## 2. Methods

The dynamic enzyme-cost flux balance analysis models a metabolic reaction network coupled with gene expression as a dynamic optimization problem. By assuming the system to be self-optimizing for growth, regulatory features of the network, which are often not known in detail, need not be explicitly included in the model. Instead, the reaction fluxes are used as decision variables for the optimization problem. We present very shortly the mathematical notation of deFBA, so that the reader can understand the problems we face when building these models.

We will use sets of indices to denote submatrices and subvectors. For instance, $S_{A,*}$ denotes the submatrix of *S* corresponding to the rows in the set *A* and all columns, while $v_{R_{x}}$ denotes the subvector of *v* with the entries at the indices in $R_{x}$. Furthermore, we use |A| to denote the number of elements of a set *A*.

The model consists of *n* species divided into four different groups:

- the set of *external species* Y, present in the environment (e.g., carbon sources, oxygen, nitrogen), with corresponding molar amounts $y\left(t\right)\in R_{\geq 0}^{\left|Y\right|}$, ∀t≥0,
- the set of *internal metabolic species* X acting as precursors for the production of biomass (e.g., ATP, NADH, amino acids), with corresponding molar amounts $x\left(t\right)\in R_{\geq 0}^{\left|X\right|}$, ∀t≥0,
- the set of *storage species* C, which save energy for later usage (e.g., starch, glycogen), with corresponding molar amounts $c\left(t\right)\in R_{\geq 0}^{\left|C\right|}$, ∀t≥0,
- the set of *macromolecules* P, which are catalytic enzymes or necessary cellular building blocks, with corresponding molar amounts $p\left(t\right)\in R_{\geq 0}^{\left|P\right|}$, ∀t≥0,

with n=|Y|+|X|+|C|+|P|, [y]=[x]=[c]=[p]=mmol.

The deFBA model is a dynamic model and hence, all variables described above are considered as functions of time. As in most constraint-based modeling frameworks for metabolism, deFBA assumes that the cell has evolved to maximize its growth in the form of maximizing total biomass at each time point in the simulation period. Thus, we use the *objective weights* $b_{i}$, which are typically identical to the molecular weights $w_{i}$, $\left[b_{i}\right]=\left[w_{i}\right]=g/\mathrm{mmol}$, for all macromolecules P to define the *objective biomass* $B_{o}$ until end-time $T_{\mathrm{end}},\;\left[T_{\mathrm{end}}\right]=h$, as

(1) $$\begin{array}{c} \int_{0}^{T_{\mathrm{end}}}B_{o}\left(t\right)\;dt=\int_{0}^{T_{\mathrm{end}}}b_{P}^{T}p\left(t\right)\;dt. \end{array}$$

Additionally, we define the *total biomass* $B_{t}\left(t\right)$ by adding the weight of the storage

(2) $$\begin{array}{c} B_{t}\left(t\right)=B_{o}\left(t\right)+w_{C}^{T}c\left(t\right). \end{array}$$

The optimization problem is constructed with the assumption that reaction rates (fluxes) $v_{i}\left(t\right)$, $[v_{i}\left(t\right)]=\mathrm{mmol}/h$, which are also time-dependent, are chosen to maximize the biomass accumulation over the simulation time $\left[0,T_{\mathrm{end}}\right]$ given the initial macromolecule amounts $p_{0}$. The objective function is thus constructed as the biomass integral

(3) $$\begin{array}{c} \max_{v(t)}\;B\left(p_{0},T_{\mathrm{end}}\right)=\max_{v(t)}\int_{0}^{T_{\mathrm{end}}}b_{P}^{T}p\left(t\right)\;dt, \end{array}$$

in which we use the objective biomass Equation (1).

Note that, although in our formulation storage species are not part of the objective biomass, the deFBA formalism does not strictly prohibit this. This means that, if for the modeled organism the storage should be part of the objective biomass, this can be incorporated. Furthermore, note that we allow some of the objective weights $b_{i}$ to be zero, in order to account, e.g., for the possibility that the modeled organism secretes enzymes that then catalyze external reactions.

As with the species we differentiate the *r* reactions into four groups

- the set of exchange and external reactions $R_{y}$, which transport matter between the cell and the environment or convert external species into each other, with corresponding fluxes $v_{R_{y}}\left(t\right)\in R^{|R_{y}|}$, ∀t≥0,
- the set of internal metabolic reactions $R_{x}$, which convert internal metabolites into each other, with corresponding fluxes $v_{R_{x}}\left(t\right)\in R^{|R_{x}|}$, ∀t≥0,
- the set of storage reactions $R_{c}$, which convert between internal metabolites and storage, with corresponding fluxes $v_{R_{c}}\left(t\right)\in R^{|R_{c}|}$, ∀t≥0,
- the set of biomass reactions $R_{p}$, which synthesize macromolecules from internal metabolite precursors, with corresponding fluxes $v_{R_{p}}\left(t\right)\in R_{\geq 0}^{|R_{p}|}$, ∀t≥0,

where $r=|R_{y}\left|+\right|R_{x}\left|+\right|R_{c}\left|+\right|R_{p}|$, and the set of all reactions R is given by $R=R_{y}\cup R_{x}\cup R_{c}\cup R_{p}$.

We note that in deFBA models each reaction $R_{p}$ is producing a biomass component, as opposed to regular FBA models, which only maximize the flux through a single biomass producing reaction.

The differential equations describing the dynamics of the species are given by the stoichiometric matrix $S\in R^{n\times r}$ as

(4) $$\frac{d}{dt}\left(\begin{array}{c} y(t)\\ x(t)\\ c(t)\\ p(t) \end{array}\right)=S\left(\begin{array}{c} v_{R_{y}}\left(t\right)\\ v_{R_{x}}\left(t\right)\\ v_{R_{c}}\left(t\right)\\ v_{R_{p}}\left(t\right) \end{array}\right)=\left(\begin{array}{c} S_{Y,R_{y}}v_{R_{y}}\left(t\right)\\ S_{X,R_{y}}v_{R_{y}}\left(t\right)+S_{X,R_{x}}v_{R_{x}}\left(t\right)+S_{X,R_{c}}v_{R_{c}}\left(t\right)+S_{X,R_{p}}v_{R_{p}}\left(t\right)\\ S_{C,R_{c}}v_{R_{c}}\left(t\right)\\ S_{P,R_{p}}v_{R_{p}}\left(t\right) \end{array}\right),$$

for all t≥0, where the entries $S_{i,j}$ give the stoichiometry of species *i* in reaction *j*.

The complexity of the problem is reduced using a quasi-steady-state approximation for the internal metabolites as

(5) $$\begin{array}{c} \frac{d}{dt}x\left(t\right)=0\Leftrightarrow S_{X,R_{y}}v_{R_{y}}\left(t\right)+S_{X,R_{x}}v_{R_{x}}\left(t\right)+S_{X,R_{c}}v_{R_{c}}\left(t\right)+S_{X,R_{p}}v_{R_{p}}\left(t\right)=0,\forall t\geq 0. \end{array}$$

Furthermore, flux constraints which are independent of enzymatic capacity can be added as

(6) $$\begin{array}{c} v_{\min}\leq v\left(t\right)\leq v_{\max},\forall t\geq 0. \end{array}$$

In flux balance analysis (FBA) [21,22], where only the part of the system corresponding to internal and exchange reactions is modeled and a static biomass objective function is maximized, these box constraints are necessary to limit the growth yield, defined as the flux through the biomass reaction. For our application, the limiting factor for the growth rate is the capacity of the enzymes to catalyze the reactions, depending on the *catalytic constants* $k_{\mathrm{cat}}$. Individual enzymes may catalyze multiple reactions. Hence, we denote the set of reactions catalyzed by the enzyme $P_{i}$ as

$$\begin{array}{c} \mathrm{cat}\left(P_{i}\right)=\left\{R_{j}\;|\;P_{i}\;\mathrm{catalyzes}\;R_{j}\right\} \end{array}$$

and constrain the reactions fluxes via

$$\begin{array}{c} \sum_{R_{j}\in \mathrm{cat}\left(P_{i}\right)}\left|\frac{v_{R_{j}}\left(t\right)}{k_{\mathrm{cat}}^{\pm R_{j}}}\right|\leq p_{i}\left(t\right),\forall t\geq 0, \end{array}$$

with the forward (backward) constant $k_{\mathrm{cat}}^{+R_{j}}$ ($k_{\mathrm{cat}}^{-R_{j}}$), $\left[k_{\mathrm{cat}}^{\pm R_{j}}\right]=h^{-1}$. Similarly, the amount of ribosome constrains the total rate of protein synthesis in the model. All these constraints can be formulated linearly as

(7) $$\begin{array}{c} H_{C}v\left(t\right)\leq H_{E}p\left(t\right),\;\forall t\geq 0, \end{array}$$

with the *capacity matrix* $H_{C}$ containing the catalytic constants and the *filter matrix* $H_{E}$ containing exactly one non-zero entry per row. An example of how to construct the matrices $H_{C}$ and $H_{E}$ for the model introduced in Section 8 can be found in the Supplements. The *enzyme capacity constraint* Inequality (7) must be satisfied at all times. Assuming any pathway from nutrients to biomass contains at least one reaction limited by an enzyme, the rate of this reaction will be limiting and thus the growth rate will be finite at all times.

In addition to enzymes and ribosomes, deFBA models also include noncatalytic biomass. These are macromolecules of the cell that fulfill no immediate catalytic activity, such as the cell wall or the membrane, but are nevertheless crucial for reproduction and their synthesis consumes cellular resources. To model this, we impose a constraint to enforce the production of a certain noncatalytic biomass component in a proportional way with the catalytic biomass. We call these species *quota compounds*. As an example, consider a quota macromolecule $P_{s}$ and assume it must make up 20% of the total biomass $B_{t}$ at any time point t≥0. We express this as

(8) $$\begin{array}{c} w_{s}p_{s}\left(t\right)\geq \phi_{s}B_{t}\left(t\right), \end{array}$$

with $\phi_{s}=0.2$. We call the according matrix formulation the *biomass composition constraint* and write

(9) $$\begin{array}{c} H_{B}\left(\begin{array}{c} c(t)\\ p(t) \end{array}\right)\leq 0,\;\forall t\geq 0. \end{array}$$

An example of $H_{B}$ for the model in Section 8 can be found in the Supplements.

Finally, since deFBA models do not include all resource and energy consuming processes in the cell, an ATP-maintenance reaction may be used to tune the model-derived growth rate and represent additional unmodeled energy sinks. An ATP-maintenance reaction hydrolyzes ATP as

$$\begin{array}{c} \mathrm{ATP}\to \mathrm{ADP}+\mathrm{Pi}. \end{array}$$

These reactions are typically enforced proportionally to the total biomass. Thus we assign each maintenance reaction $R_{m}$ a maintenance coefficient $\psi_{m}$, $\left[\psi_{m}\right]=\mathrm{mmol}/\left(g\cdot h\right)$, and write

(10) $$\begin{array}{c} v_{R_{m}}\left(t\right)\geq \psi_{m}B_{t}\left(t\right)\Leftrightarrow v\left(t\right)\geq H_{M}\left(\begin{array}{c} c(t)\\ p(t) \end{array}\right),\;\forall t\geq 0. \end{array}$$

An example of $H_{M}$ for the model in Section 8 can be found in the Supplements.

We do not include the maintenance reactions as an individual class of reactions, as we are usually only handling very few of them in comparison to other reactions. The maintenance reactions will thus typically be a subset of the metabolic reaction set $R_{x}$.

To formulate the dynamic optimization problem we need to choose initial conditions for the external species $y_{0}$, storage species $c_{0}$, and the macromolecules $p_{0}$. In many cases, one can assume that cells are adapted to achieve maximum growth rate in a certain medium in which they have been cultured before the start of the process modeled by deFBA. To obtain the biomass composition in these cases, a good strategy is to solve an RBA problem [2] with extracellular species amounts $y_{0}$ based on the preculture medium, yielding storage and macromolecule amounts $c_{0}\left(y_{0}\right)$ and $p_{0}\left(y_{0}\right)$ for optimal growth in this medium. The initial values are then set as

(11) $$\begin{array}{c} y\left(0\right)=y_{0},\;c\left(0\right)=c_{0}\left(y_{0}\right),\;p\left(0\right)=p_{0}\left(y_{0}\right). \end{array}$$

The metabolites x(t) operate in quasi steady-state (see Equation (14)) and thus do not need initial values. The complete deFBA problem then reads

(12) $$\begin{array}{cc} \max_{v(t)} & \int_{0}^{T_{\mathrm{end}}}B_{o}\left(t\right)\;dt=\int_{0}^{T_{\mathrm{end}}}b_{P}^{T}p\left(t\right)\;dt \end{array}$$

(13) $$\begin{array}{ccc} s.t.\; & \frac{d}{dt}\left(\begin{array}{c} y(t)\\ x(t)\\ c(t)\\ p(t) \end{array}\right)=S\left(\begin{array}{c} v_{R_{y}}\left(t\right)\\ v_{R_{x}}\left(t\right)\\ v_{R_{c}}\left(t\right)\\ v_{R_{p}}\left(t\right) \end{array}\right), & \forall t\geq 0 \end{array}$$

(14) $$\begin{array}{cc} \frac{d}{dt}x\left(t\right)=0, & \forall t\geq 0 \end{array}$$

(15) $$\begin{array}{cc} v_{\min}\leq v\left(t\right)\leq v_{\max}, & \forall t\geq 0 \end{array}$$

(16) $$\begin{array}{cc} H_{C}v\left(t\right)\leq H_{E}p\left(t\right), & \forall t\geq 0 \end{array}$$

(17) $$\begin{array}{cc} H_{B}\left(\begin{array}{c} c(t)\\ p(t) \end{array}\right)\leq 0, & \forall t\geq 0 \end{array}$$

(18) $$\begin{array}{cc} v\left(t\right)\geq H_{M}\left(\begin{array}{c} c(t)\\ p(t) \end{array}\right), & \forall t\geq 0 \end{array}$$

(19) $$\begin{array}{c} y\left(0\right)=y_{0},\;c\left(0\right)=c_{0},\;p\left(0\right)=p_{0} \end{array}$$

(20) $$\begin{array}{cc} y(t),\;c(t),\;p(t)\geq 0, & \forall t\geq 0 \end{array}$$

This dynamic optimization problem can be solved by discretizing time using a collocation method [8]. This way the problem is cast into a linear program (LP), which can be solved using standard commercial solvers such as CPLEX or Gurobi or open source solvers such as cvxopt [23] or the more numerically stable SoPlex [24,25,26]. The linearity of the problem is given by modeling molar amounts of species instead of concentrations, see [8] for further details. With respect to the computational and numerical details of solving such problems, we refer the reader to [8], and to [10] for a large scale example.

## 3. Model Prerequisites

The most important prerequisite for building a metabolic resource allocation model is a (genome-scale) metabolic reconstruction of the organism of interest. As we will see in the following sections, key ingredients that this reconstruction should contain are

- the reactions of central carbon metabolism,
- the reactions of the amino acid synthesis pathways,
- the pathways for the biosynthesis of precursors of structural cell components (e.g., lipids for the membrane),
- a gene-reaction mapping,
- an accurate biomass objective function (if quota compounds are to be included).

A description of how exactly to come up with such a genome-scale metabolic reconstruction is out of the scope of this article. To date, more than 2600 functional draft reconstructions have been generated [38] and many of them can be retrieved from online databases such as BioModels [30,39,40,41]. If there exists no genome-scale metabolic reconstruction for the organism of interest, but the full genome sequence of the organism is available, the protocol of Thiele et al. [15] can be followed in order to generate the metabolic network reconstruction.

One remark we would like to make is that it may not be possible to simulate a complete genome-scale deFBA model due to the size of the resulting linear program. While RBA allows simulation of steady-state resource allocation in genome-scale networks [42], the dynamic approaches like deFBA are currently constrained to smaller sizes. Networks with up to 500 metabolic reactions can be successfully simulated with deFBA as demonstrated in [10]. If the starting metabolic network is too large, tools such as the minimal network finder in [43] or procedures as described in [44,45] can be used to reduce the size of the network while keeping desired functionalities in the model. We note that, although the model formalism does not prohibit it in any way, lumping of reactions for model reduction as done in [46] is difficult within the scope of resource allocation models. The difficulty arises in deciding how far the lumping should go, but more importantly in adjusting the production cost of the lumped enzymes as well as the turnover rate. The cost adjustment difficulty is brought in particular by lumping reactions that share enzymes with other pathways.

Another key prerequisite of a deFBA model are the annotated amino acid sequences of all the genes present in the model, as we will explain next.

## 4. Building the Protein Production Reactions

Any good quality genome-scale metabolic reconstruction contains a gene-reaction mapping. Such a mapping describes which genes are involved in the catalysis of each reaction. In addition, it offers information about isoenzymes, i.e., enzymes that differ in amino acid sequence but catalyze the same reaction. In the following subsections we will explain how to make use of the gene-reaction mapping in order to construct the protein synthesis reactions for a deFBA model.

### 4.1. The Case of Enzymes Encoded by One Gene Only

To build any protein production reaction using the gene-reaction mapping the key ingredient is a database of all genes to be included in the model and their corresponding amino acid sequences. This can be obtained as a FASTA file from online databases such as Genbank [27] or UniProt [47]. A FASTA file is formatted such that it represents either nucleotide or peptide sequences using single-letter codes. The advantage of using UniProt is that, through the Java API, one can automatically access the sequences, as well as information about the Enzyme Commission number (EC number) [48] or sometimes even subunit stoichiometry for enzymes. There also exist organism-specific databases where this information can be retrieved. To give some examples, in the case of *Saccharomyces cerevisiae* one could obtain such a FASTA file also from the Saccharomyces Genome Database [17], while for cyanobacteria one could use Cyanobase [49].

To build the synthesis reaction for an enzyme encoded by exactly one gene, we look up the corresponding entry in the FASTA file, compute its amino acid count, and set the amino acids with their respective counts as reactants for the production reaction. Additional reactants are then the energy cofactors needed to grow the peptide chain: per amino acid added, one ATP is hydrolyzed into AMP and PP${}_{i}$, and two GTP molecules are hydrolyzed into GDP and P${}_{i}$ [50]. Depending on the granularity of the model, the modeler may decide to include tRNA-amino acid complexes, cofactors, or prosthetic groups in the enzyme synthesis reactions. However, this involves additional manual curation and in general it cannot be automated. As an alternative, these can be included as quota compounds as detailed in Section 5.

For small scale models and toy models where the different energy cofactors are not modeled (e.g., GTP), we recommend merging the energy requirements into a single term that is dependent on the enzyme size, as done in the example in Section 8.

Another important factor that comes into play is whether the enzyme is a monomer, i.e., if only one copy of the corresponding gene is needed to build the enzyme. Otherwise the amount of copies of the gene required to produce the enzyme has to be accounted for in the production reaction. This information can be often retrieved from the UniProt database. As an example, if the enzyme is a homotrimer, i.e., three copies of the gene are needed to build it, then the stoichiometries of the amino acids and the energy cofactors in the production reaction have to be multiplied by three. The importance of taking into account such information can be seen in the cost of producing such an enzyme. If there are alternative pathways, not taking into account these extra costs may result in the optimization approach predicting a wrong pathway choice.

### 4.2. The Case of Enzyme Complexes Encoded by Several Genes

Suppose we want to build the production reaction for an enzyme encoded by three genes as: $g_{1}$ and $g_{2}$ and $g_{3}$. In this case, we compute the amino acid counts for all three genes, add them up and use them as reactants as described in Section 4.1, while making sure we also adapt the ATP and GTP requirements. This strategy is however only correct if the peptides encoded by each gene participate in the enzyme as monomers. This is not always the case, and often peptides encoded by one gene participate in enzymes as dimers or trimers.

An example is the enzyme isocitrate dehydrogenase in yeast, composed of the gene products of YOR136W and YNL037C, where the corresponding gene products both participate as dimers. Hence the amino acid counts for each gene product should be multiplied by two and then added up when setting up the synthesis reaction (see Table A1 and Table A2 in the Appendix A) Therefore, in general, before we add up the amino acid counts for all genes, we have to multiply the counts with the factor with which they participate in the enzyme.

Information about the stoichiometry of individual peptides within enzymes is unfortunately not readily incorporated in genome-scale metabolic network reconstructions and can often only be found through extensive literature research or by querying the UniProt database.

### 4.3. The Case of Isoenzymes

Isoenzymes usually arise as a result of partial genome duplication and subsequent point mutations or insertion/deletion events in the course of evolution. They usually have different kinetic properties and are subject to different regulatory influences. Isoenzymes are important features of metabolism that allow fine-tuning of reactions rates in a way that satisfies the exact needs of the organism in different environments and at different stages of development or of the cell cycle.

Isoenzymes also play a special role in a deFBA model. To see this, let us assume we have two enzymes $e_{1}$ and $e_{2}$ that catalyze the same irreversible reaction *r*. In the deFBA model we would then build two enzyme production reactions with different amino acid requirements for the two enzymes and then the sum of the amounts of these enzymes will bound the flux through reaction *r* together with the corresponding turnover rates. For simplicity, however, we recommend that the reaction *r* is transformed into two identical reactions $r_{1}$ and $r_{2}$, each catalyzed by one of the two isoenzymes, and whose fluxes are bounded as

$$\begin{array}{cc} v_{r_{1}}\left(t\right) & \leq k_{\mathrm{cat}}^{r_{1}}p_{e_{1}}\left(t\right),\forall t\geq 0,\\ v_{r_{2}}\left(t\right) & \leq k_{\mathrm{cat}}^{r_{2}}p_{e_{2}}\left(t\right),\forall t\geq 0. \end{array}$$

Having mentioned turnover rates, it is important to keep in mind that usually isoenzymes are assigned the same Enzyme Commission (EC) number. This means that, when searching turnover rates for isoenzymes we are usually bound to find the same values in databases such as BRENDA [36], although in reality the turnover rates may be different. This is an inherent problem with EC numbers as they are mapping from a reaction to a family of enzymes catalyzing this reaction. Therefore, one can usually only find a single kcat value for a reaction even if several isoenzymes are known. Without more specific information, we are forced to use the same catalytic constant for all isoenzymes.

In this situation, the only distinguishing feature of isoenzymes from the perspective of resource allocation, is their amino acid and translation cost. However, if they do not make a significant difference in the complexity of the resulting linear program, it is recommended that all isoenzymes, including the longer ones, are represented separately in the system.

The way the isoenzyme production reactions are built depends on their gene structure, as described in Section 4.1 and Section 4.2.

### 4.4. The Ribosome

In deFBA models, the ribosome is assumed to simply have catalytic function just as any other enzyme. The amount of ribosome constrains the combined fluxes through the protein production reactions via different turnover rates.

Considering the ribosome to be an “enzyme” encoded by several genes, its production reaction can be modeled as described in Section 4.2 above. As opposed to usual enzymes for which it may be difficult to find the stoichiometry of individual peptides, the ribosomes are rather well studied and information about their composition can be found for many organisms in the Kyoto Encyclopedia of Genes and Genomes (KEGG) resource [18]. In addition to the ribosomal proteins, also the ribosomal RNA needs to be taken into account for the production reaction. Information about this can also be found in the KEGG resource.

The ribosome translation rate is a key parameter in a deFBA model and it has very high impact on the tradeoffs that govern the choice of one model behavior over another. This parameter directly affects the required ribosome fraction to sustain a certain growth rate.

Ribosome translation rates vary between prokaryotes and eukaryotes and they even vary with growth rate within the same organism [51]. They are usually measured in attached amino acids per second, and hence the efficiency of the ribosome for building different enzymes is dependent on this parameter, but also on the respective enzymes’ lengths. In general the formula for computing the $k_{\mathrm{cat}}$ of the ribosome for the production of a protein is thus given by

$$k_{\mathrm{cat}}=\frac{a}{l},$$

where *a* is the ribosome rate in amino acids per unit of time, and *l* is the length of the protein in amino acids. For instance, if we consider the translation of one enzyme of 100 amino acids by a bacterial ribosome with a rate of 15 amino acids per second then, assuming the enzyme does not compete with other proteins for the ribosome, the enzyme will be translated with a catalytic constant of $15\;s^{-1}/100=540\;h^{-1}$, i.e., a maximal rate of 540 enzyme units per hour and ribosome unit.

### 4.5. Compartmentalization

Eukaryotic cells, as opposed to prokaryotic ones, are usually compartmentalized (into cytosol, mitochondrion etc.). This compartmentalization plays a role in the way enzymes are built, in the sense that there may be identical enzymes that are active in the cytosol as well as in the mitochondrion for example. In this case, two production reactions should be used, one for each compartment, since an enzyme that is in the cytosol cannot catalyze a reaction in the mitochondrion.

We give an example from the Yeast 6 network [52]. In this metabolic network there are two fumarase reactions annotated—one cytosolic and one mitochondrial, both with the same gene association—YPL262W. Therefore, in the deFBA model, we must have two fumarase enzymes, cytosolic-fumarase and mitochondrial-fumarase. These enzymes will have each their own production reaction and will only catalyze reactions in their respective compartments.

## 5. Setting Up Quota Compounds

Although the catalytic biomass is the main part of the model that is responsible for the autocatalytic cycle, there are several noncatalytic components (which we call quota compounds) that are also needed in a full cell model. Examples are DNA, RNA, cell wall or membrane. Without accounting for the growth and duplication of these components we would be neglecting a significant biosynthetic energy requirement.

As explained in Section 2, in the model the production of these compounds is ensured by using the biomass composition constraint (9). However, the question then arises: what are appropriate biomass fractions $\phi_{s}$ that we should impose for these compounds?

### 5.1. Constructing Noncatalytic Biomass Requirements

A good place to look for the total biomass fractions that should be dedicated to quota compounds is the biomass reaction of the metabolic network reconstruction. The stoichiometric coefficients for the substrates of this artificial reaction describe the average composition of the modeled cell. To better understand this, let us take a look at the biomass reaction of the Yeast 6 model [52], which we have reproduced in Table A3 in the Appendix B.

We observe there the main biomass components: proteins (in the form of charged transfer RNAs), storage (glycogen and trehalose), DNA (dAMP, dCMP, dGMP, dTMP), RNA (AMP, CMP, GMP, UMP), cell wall (mannan and β-D-glucan), membrane (lumped lipid), other small molecules, and the ATP energy needed for polymerization. For ease of understanding later on, we denote the biomass reaction by $R_{b}$, the indices of biomass components (reactants of biomass reaction) as $I_{bio}$, and the indices of biomass byproducts (products of biomass reaction) as $I_{bp}$. Then the biomass reaction has the form

$$\begin{array}{c} \sum_{i\in I_{bio}}S_{i,R_{b}}X_{i}\to 1\;g\;\mathrm{biomass}+\sum_{i\in I_{bp}}S_{i,R_{b}}X_{i}, \end{array}$$

where $X_{i}$ denotes the *i*-th internal metabolite and $[S_{i,R_{b}}]=\mathrm{mmol}/g$.

In general, the reactant stoichiometries for the biomass reaction are chosen such that, when weighted by the corresponding molecular weights $w_{i}$, they add up to 1, i.e.,

$$\begin{array}{c} \sum_{i\in I_{bio}}S_{i,R_{b}}w_{i}-\sum_{i\in I_{bp}}S_{i,R_{b}}w_{i}=1. \end{array}$$

To reduce the number of quota compounds in the model, we lump these together and build spontaneous reactions that produce the artificial merged quota compounds such as generic cell wall.

As an example, all charged transfer RNAs (whose indices we denote by $I_{aa-tRNA}\subset I_{bio}$) would be consumed to produce one merged protein quota metabolite, and release all the uncharged tRNAs (indices denoted by $I_{tRNA}\subset I_{bp}$). In setting up this reaction, we should make sure that we adjust the stoichiometric coefficients in such a way that, multiplied with the corresponding amino acids’ molecular weights, they add up to one, i.e., we need to divide them by

$$\phi_{\mathrm{protein}}=\sum_{i\in I_{aa-tRNA}}S_{iR_{b}}w_{i}.$$

Thus, the protein quota building reaction will read

$$\begin{array}{c} \sum_{i\in I_{aa-tRNA}}\frac{S_{i,R_{b}}}{\phi_{\mathrm{protein}}}X_{i}+\frac{S_{ATP,R_{b}}}{\phi_{\mathrm{protein}}}\mathrm{ATP}+\frac{S_{H_{2}O,R_{b}}}{\phi_{\mathrm{protein}}}H_{2}O\\ \to 1\;\mathrm{protein}+\sum_{i\in I_{tRNA}}\frac{S_{i,R_{b}}}{\phi_{\mathrm{protein}}}X_{i}+\frac{S_{ADP,R_{b}}}{\phi_{\mathrm{protein}}}\mathrm{ADP}+\frac{S_{phosphate,R_{b}}}{\phi_{\mathrm{protein}}}\mathrm{phosphate}+\frac{S_{H^{+},R_{b}}}{\phi_{\mathrm{protein}}}H^{+}. \end{array}$$

Therefore, for Yeast 6 the corresponding quota production reaction takes a form as shown in Table A4 in the Appendix B. Please note that we have also added to this reaction the necessary ATP needed for polymerization, which is the fraction $\phi_{\mathrm{protein}}=0.466298$ of the total ATP consumed in the original biomass reaction.

After setting up this reaction, the amount of protein quota would be $\phi_{\mathrm{protein}}=0.466298$. However, of these proteins, some are modeled explicitly as enzymes, and in the next section we will see how to adjust the $\phi_{\mathrm{protein}}$ to only require the proteins that are not modeled as enzymes or ribosome. For the rest of the quota compounds (DNA, RNA, cell wall, membrane, other small molecules) we would proceed in a similar fashion as for the proteins, with the sole difference that their $\phi_{i}$ would not need to be adjusted once computed.

### 5.2. The Case of Noncatalytic Proteins

The noncatalytic proteins quota poses a special case because, in the typical biomass reactions, we only have one component called ’protein’, which encompasses all protein content present in one gram dry weight of cells. However, we need to distinguish in a deFBA model between proteins that are explicitly accounted for as enzymes, and not explicitly modeled proteins that should be put into the protein quota compound.

One way to do this is to find a (genome-scale) quantitative proteomics dataset. If not already scaled, we normalize the protein amounts in the dataset to add up to 1. We observe that in this dataset we find two types of proteins corresponding to our model: those included explicitly in the model, which sum up to a fraction $f_{e}$, and those not present explicitly in our model, that we call quota proteins, and which sum up to $1-f_{e}$ after the normalization of the dataset.

Since we want to adapt the protein quota to only account for the noncatalytic proteins, we adjust $\phi_{\mathrm{protein}}$ by multiplying it with the fraction $1-f_{e}$ of noncatalytic proteins in the dataset.

An important issue that arises here is the growth rate at which the cells were growing when used for the quantitative proteomics measurement. Several studies show that, for instance, the total amount of ribosomes grows linearly with the growth rate and that partitioning of proteome strongly varies with growth rate and growth conditions [53,54,55]. Since deFBA models an autocatalytic system where typically exponential growth is the predicted optimal solution, quantitative proteomics datasets from exponentially growing cultures should be used if available.

Last but not least, we note that the production reaction for noncatalytic protein quota is not spontaneous. It is catalyzed by the ribosome and competes this way for the ribosome with the enzyme production reactions. Thus, we have to compute a turnover rate for it as described in Section 4.4 above, by using the sum of the stoichiometric coefficients of the amino acids (or of the amino acid-tRNA complexes) as the protein length. The unit of this turnover rate however is not h${}^{-1}$, but ${\mathrm{mmol}}^{-1}\cdot h^{-1}$ because of how the lumping reaction is set up.

### 5.3. Storage

Besides the catalytic macromolecules and the quota described above, most microorganisms also produce storage macromolecules, cf. Section 2. For some of them, like cyanobacteria, the storage is essential to survive the night period, when no energy from the sun is present. For others, like yeast, the storage is used to survive through periods of starvation or as an energetic reserve for the production of new enzymes and transporters as a consequence of sudden changes in the environment. Therefore, we strongly advise to include the storage macromolecules in the resource allocation model if it is going to be used for dynamic simulations. Looking at the biomass reaction of Yeast 6 in Table A3 in the Appendix B, we observe that glycogen and trehalose are included as reaction substrates. In fact, these are storage macromolecules in yeast and should directly be included as such in a resource allocation model.

There are a number of possible choices how to take the specific role of storage molecules into account for these models. If there is evidence that a certain fraction of storage molecules is always present in the situations to be described by the model, one could in principle include them as quota compounds with a biomass composition constraint. However, this has to be done with caution because this constraint will then prohibit the model from using up storage molecules in a situation where it would actually be needed. Also, for most cases, we recommend not to include the weight of storage in the biomass objective function (see Equations (2) and (3)), because inclusion of storage may lead to unrealistic growth modes in some situations as discussed in [56]. Nevertheless, depending on the usage of storage in the model, one can of course add the storage molecules to the objective function if needed. In both cases the storage should be included in the total biomass for scaling of biomass dependent constraints, e.g., maintenance reactions.

## 6. Assigning Reaction Turnover Rates

Turnover rates are necessary parameters in a resource allocation model. They are involved in the capacity constraints on reaction fluxes using the amount of their catalyzing enzymes.

Such turnover rates can be derived from experimental data as explained in [57]. Alternatively, a recent study has shown that turnover rates reported in online databases are a good enough approximation of in vivo turnover rates [58].

The two main databases for retrieving turnover rates are BRENDA [36] and SABIO-RK [37]. While BRENDA stores both manually curated as well as text mining data, SABIO-RK only offers data that was either manually extracted from the literature or directly submitted by experimenters. As a result, BRENDA offers a larger amount of turnover rates than SABIO-RK. On the other hand, the text mining entries may not have the same quality as the manually curated ones and the incorporation of these values in resource allocation models should be done with care and if possible these values should be manually checked. Both databases have automated retrieval options.

Some simple rules of thumb for retrieving turnover rates from these databases are that one should filter for wild type, non-recombinant values, and, if possible, should make sure that the measurements were done at (nearly) physiological pH. This typically narrows down the results significantly such that alternatives can be investigated or a median of the remaining values can be used, in a similar fashion as explained below.

Although large amounts of biochemical data are now available, usually not all turnover rates for the organism of interest can be found. We recommend that in this case, if turnover rates for a given enzyme from other organisms are found, that these should be used. Moreover, it is important to perform a sensitivity analysis to check the influence of these unknown parameters on the results.

The question then arises: which of the available other organism turnover rates should be used? Should it be a mean or a median of all found turnover rates for the respective enzyme, or the turnover rate from the organism that has the most sequence similarity with the target organism within that protein?

To answer this question, we have automatically retrieved wild type turnover rates from the BRENDA database of all enzymes for three organisms: *Saccharomyces cerevisiae*, *Escherichia coli*, and *Bacillus subtilis*. In a second iteration, we retrieved turnover rates of all enzymes from all other organisms, excluding the organism of interest, and computed the mean, median, and best sequence match with the organism of interest $k_{\mathrm{cat}}$ on a per enzyme basis. The best sequence match was obtained by computing the alignment score using the Needleman-Wunsch algorithm [59] with the BLOSUM62 scoring matrix [60]. We computed the Pearson correlation coefficients between the logarithms of $k_{\mathrm{cat}}$ values from the organism of interest and the logarithms of the mean, median and best sequence match $k_{\mathrm{cat}}$ values obtained from other organisms. Only values corresponding to the same catalyzed reaction were compared. The resulting correlation coefficients are displayed in Table 2.

We observe that, in the cases we have analyzed, the medians of all turnover rates enzyme-wise is the best approximation for the actual turnover rates in the organism of interest. Moreover, the order of magnitude correlation coefficients are very high and the *p*-values we get are all in the order of $10^{-14}$ or lower, indicating that indeed these median turnover rates from other organisms are good enough approximations of the real $k_{\mathrm{cat}}$ values, if no specific data is available for the organism of interest.

**Figure 2:**
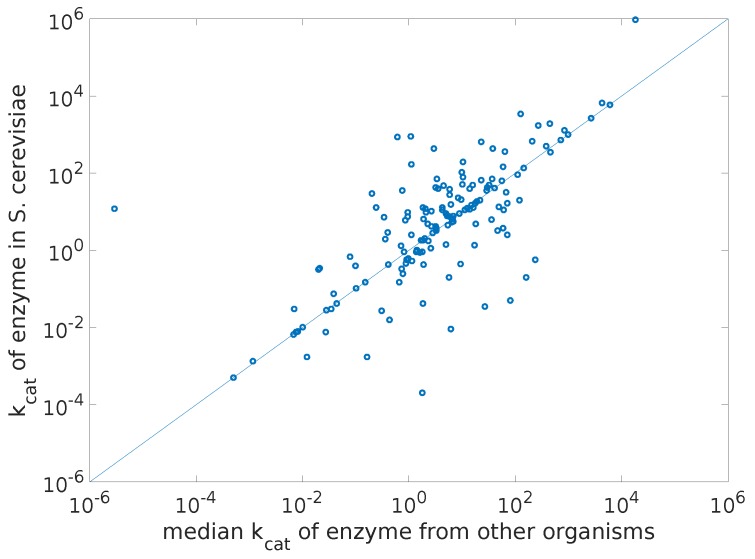
Turnover rates in yeast versus the median $k_{\mathrm{cat}}$ values from other organisms.

To give an idea of the spread of the turnover rate data, we show in Figure 2 a plot of the $k_{\mathrm{cat}}$ values in yeast versus the median $k_{\mathrm{cat}}$ values from other organisms.

## 7. Validating the Model Using Experimental Data

Once the model has been constructed, a first step before further investigations is its validation using experimental data. This can stretch from fairly basic matching of growth rates obtained from batch experiments to matching of reaction fluxes if measurements of these are available.

In general, the growth rate obtained in the model under constant conditions should provide an upper bound on the growth rate measured in the lab, since the model gives the optimal behavior of the metabolism, which may not always be observed in the lab. To check that this is the case, we need data from an exponentially growing batch culture of the organism of interest at saturating nutrient concentrations. In this case, it is sufficient to compute the growth rate of the culture $\mu_{\exp}$ as the slope of the logarithm of the optical density (OD) measurements versus time, as in Figure 1f. In addition, we also need to compute the instantaneous growth rate of the model (under the same conditions as in the experiments), which we define as

(21) $$\mu_{\mathrm{model}}\left(t\right):=\frac{1}{t_{i+1}-t_{i}}\ln\left(\frac{B_{t}\left(t_{i+1}\right)}{B_{t}\left(t_{i}\right)}\right),\;\forall t\in \left(t_{i},t_{i+1}\right).$$

We observe that, if nutrients are saturating, $\mu_{\mathrm{model}}\left(t\right)$ is constant, and hence we will refer to it in this case as simply $\mu_{\mathrm{model}}$.

If $\mu_{\mathrm{model}}$ is smaller than $\mu_{\exp}$, the problem lies very likely in the $k_{cat}$ values and these should then be checked manually. If this is not the case, $\mu_{\mathrm{model}}$ can be tuned to $\mu_{\exp}$ by forcing a biomass dependent flux through a suited maintenance reaction, e.g., hydrolysis of ATP into ADP and phosphate. Note that the flux we need to force through maintenance strongly depends on how detailed the model is and how much lumping of reactions has been done. We therefore cannot provide an order of magnitude approximation for how much flux should be forced. Modelers should however be aware that the need for a large ATP maintenance forced flux to match experimental growth rates is an indication of model errors or poor model quality. Using a maintenance reaction makes sense also from a biological perspective, since one can usually not claim that the constructed resource allocation model covers all energy expending processes in an actual cell, so such an ATP maintenance reaction serves the purpose of modeling this remaining energy expenditure.

## 8. The SBML Representation of a Resource Allocation Model

For metabolic network models used together with constraint-based modeling, it is standard to define them in the systems biology markup language (SBML), an XML-based way of representing models (http://sbml.org/Main_Page). However, there is so far no specific way of representing resource allocation models, which come with several extra ingredients in addition to the metabolic network part. Therefore, in this section we propose a way of making use of the existing SBML capabilities for representing resource allocation models [13]. We illustrate this proposal using a toy resource allocation model listed in Table 3 for which we attach the SBML representation in the Supplement.

### 8.1. Compartments

We keep any compartments present from the original reconstruction of the metabolic network. If possible, we place species objects for the additional gene products in the compartments in which the corresponding enzymes are acting. This means, e.g., that enzymes are located where the reactions they catalyze are happening. Species without any real physical location in the model, e.g., lumped species, can be placed arbitrarily in any compartment.

Note that the compartments are not used in the formulation of the deFBA problem, besides the identification of external species.

### 8.2. Species

As postulated by the SBML standard, each species object must contain the attributes:

- id
- compartment
- constant (true or false)
- boundaryCondition (true or false)
- hasOnlySubstanceUnits (true or false)
- initialAmount

All species in the model also contain a ram:species annotation. We distinguish between limiting extracellular metabolites, for which the amount changes by cellular uptake (as N${}_{1}$ and N${}_{2}$ in the toy model), nonlimiting extracellular metabolites, where the amount is assumed to be unchanged by cellular uptake (as O${}_{2}$ in the toy model), intracellular metabolites (N, AA, ATP), storage (Stor), and biomass components (ETrans1, ETrans2, EMetab1, EMetab2, EStor, S, R).

As per SBML specifications, a boundaryCondition value of true means a differential equation derived from the reaction definitions should not be generated for the species. Thus, in RAM, the nonlimiting extracellular species should have boundaryCondition = “true” as the amount of species at the systems boundary cannot be changed by the reactions, while all other species should have boundaryCondition = “false”. The mandatory attribute constant is used in RAM, as per SBML definition, to specify whether a species amount is assumed to be changing or not in a deFBA simulation. With the deFBA approach this can only be constant = “true” for nonlimiting boundary species. Still, these species should only have constant = “false” if they are changed by extracellular processes such as oxygen through gas exchange. All other species will always have constant = “false”.

In order to generate the mathematical model for the deFBA problem in terms of molar amounts, each external, storage, and biomass species element must be assigned an initial value, either by setting the initialAmount attribute and hasOnlySubstanceUnits = “true”, or by setting the initialConcentration attribute, hasOnlySubstanceUnits = “false”, and specifying the size attribute of the respective compartment. Other mathematical models may require less information, for example for RBA it would be sufficient to specify the amounts or concentrations of the external species. We show in Table 4 examples on how to set the mandatory species fields for each metabolite type.

In addition to the required SBML fields listed above, each species must have a RAM annotation ram:speciesType specifying its type. We distinguish between the species types

- “extracellular”, which can be either limiting or nonlimiting extracellular species
- “metabolite”, which are species that obey the quasi-steady-state approximation Constraint (5)
- “storage”, which represent the storage species C
- “enzyme”, which represent species P with catalytic role involved in Constraint (7)
- “quota”, which represent quota species P that are enforced using Constraint (9)

The biomass species have additionally an annotation field ram:molecularWeight for storing their molecular weight $w_{i}$, an annotation field ram:objectiveWeight for their objective weight $b_{i}$, and an annotation field ram:biomassPercentage for their the biomass percentage $\phi_{i}$. The ram:biomassPercentage attribute contains the fractions of the quota components (8) that need to be enforced at each time point.

Below we show the annotation fields for the ribosome and the structural component as examples.

Ribosome R:

		
<species id="R" name="Ribosome" compartment="cytosol" initialAmount="0.03364"
constant="false" boundaryCondition="false" hasOnlySubstanceUnits="true">
  <annotation>
              <ram:RAM xmlns:ram="https://www.fairdomhub.org/sops/304">
                <ram:species ram:molecularWeight="weight_R" ram:objectiveWeight="weight_R"
                ram:biomassPercentage="zero" ram:speciesType="enzyme"/>
              </ram:RAM>
            </annotation>
          </species>


Structural component S:


          <species id="S" name="Structural biomass component" compartment="cytosol"
          initialAmount="0.7499" constant="false" boundaryCondition="false"
          hasOnlySubstanceUnits="true">
            <annotation>
              <ram:RAM xmlns:ram="https://www.fairdomhub.org/sops/304">
                <ram:species ram:molecularWeight="weight_S" ram:objectiveWeight="weight_S"
                ram:biomassPercentage="bp_S" ram:speciesType="quota"/>
              </ram:RAM>
            </annotation>
          </species>


Note that “weight_R”, “weight_S”, “zero”, “bp_S” are ids of parameters defined in the list of parameters of the SBML model.

#### 8.2.1. Guideline to Ensure Uniqueness of Macromolecule IDs

There are some enzymes that can act in different compartments of the cell, cf. Section 4.5. An example is fumarase, which catalyzes reactions both in the cytosol and in the mitochondrion in yeast. While we include the respective compartments for the species in their description, a potential error is to give both enzymes the same id, either in their species or their fbc:geneAssociation representation. Hence, we suggest to name enzymes in a specific pattern combining, e.g., their name and their respective location.

If the enzyme is acting in only one compartment we choose its id in the format “Main_id_[acting-compartment]”. If the enzyme is a transporter between two compartments we choose “Main_id_[compartment1]_[compartment2]”. If the enzyme is translated from only one gene (e.g., ETrans1), this represents the main id. For enzyme complexes made of multiple gene products we suggest simply using “E[id(s) of catalyzed reactions]” as main id (e.g., ETrans2). Of course, the user can choose these ids freely, but following these suggestions can help with easier evaluation of the model in deFBA implementations.

### 8.3. Gene Products

Each catalytic macromolecule is not only present as a species, but also as a gene product, with the fields fbc:id, fbc:label, and fbc:associatedSpecies. The fbc:id must be unique among the fbc:geneProduct elements. Hence, we suggest using the same naming conventions as with the macromolecules. Following the Flux Balance Constraints package version 2 specifications, the fbc:label field is currently unused. Hence, we suggest to insert the recipe for the creation of the enzyme(-complex) in this attribute. Lastly, the fbc:associatedSpecies attribute contains the id of the biomass species associated with this gene product and used for bounding the flux. An example is ETrans2 from the toy model in Table 3:

		
<fbc:geneProduct fbc:id="Etrans2" fbc:label="1*GTRANS2 AND 1*GTRANS3"
fbc:associatedSpecies="Etrans2"/>


### 8.4. Reactions

#### 8.4.1. General Reaction Definition

In the SBML specification, all reaction objects must contain the fields:

- id
- reversible (true or false)
- fast (false)
- listOfReactants (may be empty)
- listOfProducts (may be empty)

Depending on their type, reactions also include a fbc:geneProductAssociation and a ram:reaction annotation. Additionally, we recommend adding the EC number, if known, to the reactions in the form of a MIRIAM annotation [61]:


<rdf:RDF xmlns:bqbiol="http://biomodels.net/biology-qualifiers/"
 xmlns:rdf="http://www.w3.org/1999/02/22-rdf-syntax-ns#">
  <rdf:Description rdf:about="#thx1138">
    <bqbiol:isVersionOf>
       <rdf:Bag>
         <rdf:li rdf:resource="http://identifiers.org/ec-code/2.7.1.17"/>
       </rdf:Bag>
    </bqbiol:isVersionOf>
  </rdf:Description>
</rdf:RDF>


The id must be unique for each reaction. For reactions producing biomass we recommend starting the id with “synth_” for easier reading and reduced chance of assigning the same id multiple times. Furthermore, this makes it easier to distinguish between the reactions from the original metabolic network model and the deFBA additions. The fast attribute will be removed in SBML Level 3 Version 2 (L3V2). Hence, we set it to “false” for now and we will delete it all together in an upcoming version once SBML L3V2 is released.

The fbc:geneProductAssociation is used to map catalysis relationships between the enzymes (which are also gene products, see above) and the reactions. The fbc:geneProductAssociation supports the inclusion of multiple fbc:geneProductRef elements connected by fbc:and and fbc:or nodes. While this can certainly be useful for certain model types, we decided to create the complex enzymes as stated above to eliminate the fbc:and connections and save the gene codes in the fbc:labels. Instead of including fbc:or elements for isoenzymes, we copy the catalyzed reaction until each reaction is catalyzed by exactly one fbc:geneProductRef (cf. Section 4.3). This way we ensure a unique interpretation of the SBML file and can easier build the deFBA model. All reactions without a fbc:geneProductAssociation are considered spontaneous (e.g., the ATP maintenance reaction), and hence their rates are not constrained by any enzyme.

The forward and reverse $k_{\mathrm{cat}}$ values for each reaction can be found in the ram:reaction annotation, in the attributes ram:kcatForward and ram:kcatBackward respectively. All irreversible reactions must have their reversible flag set to “false” and thus their ram:kcatBackward must be set to “zero”. Typically, the values in the kcatForward and kcatBackward are defined as parameters, and in these fields the ids of the respective parameters are stored, as in the examples below.

#### 8.4.2. The Maintenance Reactions

Lastly, we explain how to handle maintenance in the SBML representation. Maintenance reactions are typically part of metabolic resource allocation models, since these models do not account for all energy expenditures of the cell. The maintenance reactions we consider are all biomass-associated, i.e., the flux forced through them is dependent on the total biomass at each time point using a fixed coefficient $\psi_{m}$ as described in the Inequality (10). Thus, we add an attribute ram:maintenanceScaling inside the ram:reaction field, which specifies the coefficient $\psi_{m}$ for each reaction. For typical reactions this field is “zero” as in the case of the metabolic reaction


<reaction id="Metab1_2" reversible="false" fast="false">
  <annotation>
    <ram:RAM xmlns:ram="https://www.fairdomhub.org/sops/304">
      <ram:reaction ram:kcatForward="kcat2" ram:kcatBackward="zero"
      ram:maintenanceScaling="zero"/>
    </ram:RAM>
  </annotation>
  <fbc:geneProductAssociation fbc:id="Emetab2">
    <fbc:geneProductRef fbc:geneProduct="Emetab2" />
  </fbc:geneProductAssociation>
  <listOfReactants>
    <speciesReference species="N" stoichiometry="1" constant="true"/>
  </listOfReactants>
  <listOfProducts>
    <speciesReference species="AA" stoichiometry="1" constant="true"/>
    <speciesReference species="ATP" stoichiometry="1" constant="true"/>
  </listOfProducts>
</reaction>


For the maintenance reaction we have the representation


<reaction id="Maintenance" reversible="false" fast="false">
  <annotation>
    <ram:RAM xmlns:ram="https://www.fairdomhub.org/sops/304">
      <ram:reaction ram:kcatForward="zero" ram:kcatBackward="zero"
      ram:maintenanceScaling="main"/>
    </ram:RAM>
  </annotation>
  <listOfReactants>
    <speciesReference species="AA" stoichiometry="50" constant="true"/>
    <speciesReference species="ATP" stoichiometry="60" constant="true"/>
  </listOfReactants>
  <listOfProducts>
  </listOfProducts>
</reaction>


## 9. Discussion

Dynamic resource allocation models have emerged in recent years as a means of extending the predictive capabilities of constraint-based models. Such resource allocation models allow investigating how dynamics of the extracellular environment are reflected inside the metabolism in the form of cost-benefit tradeoffs of active pathways. This is achieved by extending FBA models in two directions: (i) accounting for the costs of producing enzymes before these can be used to catalyze their corresponding reactions; and (ii) taking a dynamic perspective where the levels of these enzymes change over time in response to changes in the environment.

Such models have a larger predictive power and can predict complex biological behaviors such as the catabolite repression as demonstrated in [8]. Moreover, a recent genome-scale dynamic resource allocation study [10] shows that these models can be used to understand the optimality of glycogen accumulation patterns in cyanobacteria. Such bacteria live in a constantly changing environment governed by the dynamics of sun light availability. By using dynamic resource allocation models, it was possible to develop a manifest hypothesis on the biology of cyanobacteria: that their metabolism is coordinated according to a temporal program that evolved to maximize growth in a diurnal environment, and that the circadian clock is the regulatory mechanism that modulates the transcriptional program of the cell to achieve this metabolic optimum.

Although their large predictive power has already been demonstrated, it is not possible to use resource allocation models for studying all biological processes connected to the metabolism. For instance, such models always assume optimality of growth. A scenario where this may not be an accurate assumption is starvation, when the objective changes more towards maximizing survival rather than growth. Moreover, starvation triggers different kinetics of nutrient uptake, where stochastic effects and substrate affinity may play a role. It is, therefore, no longer sufficient to consider only upper bounds on uptake rates based on enzyme amount and turnover rate, as done in resource allocation models.

Another limitation of such models may also lie in the fact that, so far, they do not incorporate any kind of regulatory logic that may also have an impact on the metabolic strategies, in addition to the resource tradeoffs. Some resource allocation models such as ME models indeed go a step beyond modeling translation costs and also model transcription. Others, like deFBA or RBA, include such costs in the form of biomass composition constraints and maintenance.

So far, there exist ME models of *E. coli* [5] and *T. maritima* [4], an RBA model of *B. subtilis* [2,57], and a deFBA model of *S. elongatus* [10]. Moreover, we can mention software packages for handling ME models [6] and deFBA models (Python implementation can be found at: https://bitbucket.org/hlindhor/defba-python-package; MATLAB implementation is available at https://github.com/alexandra-m-reimers/deFBA). We therefore felt there is a need in the community for a guideline for building such models as well as a means to exchange them without losing information.

Therefore, we have provided in this article a list of ingredients of such models, together with links to the relevant databases where parameters can be found. Every step of this guideline can be automated except for (i) production of enzyme complexes for which the subunit stoichiometries cannot yet be automatically retrieved from online databases; (ii) adjustment of protein quota requirements, which needs a quantitative proteomics dataset; and (iii) maintenance requirements for adjusting growth rate.

With respect to exchanging metabolic resource allocation models, we have provided here a specification that is based on SBML and the flux balance constraints package. This specification covers deFBA and RBA models and we believe that it can be extended to also incorporate ME models.

We believe that our contribution will help extend the use and number of metabolic resource allocation models as well as exchange of these models among researchers.

## Figures and Tables

**Table 1:** Databases where the necessary information needed to build a metabolic resource allocation model can be found.

Resource	Link	Reference
**Annotated genome sequences**		
Genbank	https://www.ncbi.nlm.nih.gov/genbank/	Benson et al. [27]
UniProtKB	http://www.uniprot.org/	The UniProt Consortium [28]
**Metabolic network reconstructions**		
BiGG	http://bigg.ucsd.edu/	King et al. [29]
BioModels	https://www.ebi.ac.uk/biomodels-main/	Juty et al. [30]
ModelSEED	http://modelseed.org/	Devoid et al. [31]
KEGG	http://www.genome.jp/kegg/pathway.html	Kanehisa and Goto [18]
Pathway Tools	http://bioinformatics.ai.sri.com/ptools/	Karp et al. [32]
**Enzyme subunit stoichiometry**		
UniProtKB	http://www.uniprot.org/	The UniProt Consortium [28]
**Ribosome composition**		
Ribosomal Protein Gene Database	http://ribosome.med.miyazaki-u.ac.jp/	Nakao et al. [33]
KEGG	http://www.genome.jp/kegg/	Kanehisa and Goto [18]
**Quantitative proteomics datasets**		
MaxQuant	http://maxqb.biochem.mpg.de/mxdb/	Schaab et al. [34]
Proteomaps	https://www.proteomaps.net/index.html	Liebermeister et al. [35]
**Turnover rates**		
BRENDA	http://www.BRENDA-enzymes.org/	Schomburg et al. [36]
SABIO-RK	http://sabio.villa-bosch.de/	Wittig et al. [37]

**Table 2:** Pearson correlation coefficients between the logarithms of $k_{\mathrm{cat}}$ values from the organism of interest and the logarithms of the mean, median and best sequence match $k_{\mathrm{cat}}$ values obtained from other organisms.

Organism	Median	Mean	Best Sequence Match
*Saccharomyces cerevisiae*	0.701	0.650	0.526
*Escherichia coli*	0.808	0.756	0.606
*Bacillus subtilis*	0.762	0.708	0.679

**Table 3:** List of species, reactions, and catalysis relationships for the toy model whose SBML representation can be found in the Supplement. Reaction 6 is the maintenance reaction and is considered spontaneous. Reaction 7 has different turnover rates for the forward (f) and reverse (r) directions. ETrans2 is a transporter complex. S is a structural quota component and R is the ribosome.

External Metabolites:		N${}_{1}$, N${}_{2}$, O${}_{2}$		
Internal Metabolites:		N, AA, ATP		
Macromolecules:		Stor, ETrans1, ETrans2, EMetab1, EMetab2, EStor, S, R		
**No.**	**Reactions**		**Catalysed By**	**Turnover Rate**
1	N${}_{1}$ + O${}_{2}$	↔ N	ETrans1	1800
2	N${}_{2}$ + O${}_{2}$	↔ N	ETrans2	2400
3	N	→ AA + ATP	EMetab1	2000
4	N	→ AA + ATP	EMetab2	2500
5	N	→ AA + 2 ATP	EMetab2	2000
6	50 AA + 60 ATP	→*⌀*		
7	200 AA + 300 ATP	↔ Stor	EStor	f: 25, r: 30
8	100 AA + 400 ATP	→ ETrans1	R	10
9	160 AA + 640 ATP	→ ETrans2	R	6.25
10	200 AA + 800 ATP	→ EMetab1	R	5
11	160 AA + 640 ATP	→ EMetab2	R	6.25
12	150 AA + 500 ATP	→ EStor	R	5
13	1500 AA + 200 ATP	→ S	R	10
14	1000 AA + 4000 ATP	→ R	R	1

**Table 4:** Examples for setting species fields for each species type.

	ID	Compartment	Constant	Boundary Condition	Has Only Substance Units	Initial Amount
N${}_{1}$	N1	external	false	false	true	10
O${}_{2}$	O2	external	true	true	true	10
N	N	cytosol	false	false	true	0
Stor	Stor	cytosol	false	false	true	0
R	R	cytosol	false	false	true	0.03364

## Supplementary Materials

The following are available online at www.mdpi.com/2218-1989/7/3/47/s1, Supplementary S1: A protocol for generating and exchanging (genome-scale) metabolic resource allocation models; Supplementary SBML S2: resalloc.xml

## Appendix A. Synthesis of Enzyme Complexes Composed From Several Gene Products

**Table A1:** Amino acid counts for the gene products involved in the synthesis of isocitrate dehydrogenase in yeast.

	YOR136W	YNL037C
Ala	29	29
Arg	18	18
Asn	21	19
Asp	15	18
Cys	3	0
Gln	7	8
Glu	19	22
Gly	28	32
His	7	10
Ile	29	32
Leu	20	26
Lys	34	28
Met	4	8
Phe	13	12
Pro	20	13
Ser	33	20
Thr	30	30
Trp	1	2
Tyr	9	8
Val	29	25

**Table A2:** Synthesis reaction for producing the enzyme isocitrate dehydrogenase in the Yeast 6.06 model. The two gene products that form this enzyme participate in the complex as dimers. Therefore, the counts in Table A1 are multiplied by two and added up for the synthesis reaction.

Reactants	Stoichiometry	Products	Stoichiometry
Ala-tRNA(Ala)	116	tRNA(Ala)	116
Arg-tRNA(Arg)	72	tRNA(Arg)	72
Asn-tRNA(Asn)	80	tRNA(Asn)	80
Asp-tRNA(Asp)	66	tRNA(Asp)	66
Cys-tRNA(Cys)	6	tRNA(Cys)	6
Gln-tRNA(Gln)	30	tRNA(Gln)	30
Glu-tRNA(Glu)	82	tRNA(Glu)	82
Gly-tRNA(Gly)	120	tRNA(Gly)	120
His-tRNA(His)	34	tRNA(His)	34
Ile-tRNA(Ile)	122	tRNA(Ile)	122
Leu-tRNA(Leu)	92	tRNA(Leu)	92
Lys-tRNA(Lys)	124	tRNA(Lys)	124
Met-tRNA(Met)	24	tRNA(Met)	24
Phe-tRNA(Phe)	50	tRNA(Phe)	50
Pro-tRNA(Pro)	66	tRNA(Pro)	66
Ser-tRNA(Ser)	106	tRNA(Ser)	106
Thr-tRNA(Thr)	120	tRNA(Thr)	120
Trp-tRNA(Trp)	6	tRNA(Trp)	6
Tyr-tRNA(Tyr)	34	tRNA(Tyr)	34
Val-tRNA(Val)	108	tRNA(Val)	108
ATP	1458	AMP	1458
GTP	2916	GDP	2916
H${}_{2}$O	4374	diphosphate	1458
		phosphate	2916
		H${}^{+}$	4374
		isocitrate dehydrogenase	1

## Appendix B. Biomass and the Protein Quota Synthesis Reactions

**Table A3:** Biomass reaction of the Yeast 6.06 model.

Reactants	Stoichiometry	Products	Stoichiometry
Ala-tRNA(Ala)	0.4588	tRNA(Ala)	0.4588
Arg-tRNA(Arg)	0.1607	tRNA(Arg)	0.1607
Asn-tRNA(Asn)	0.1017	tRNA(Asn)	0.1017
Asp-tRNA(Asp)	0.2975	tRNA(Asp)	0.2975
Cys-tRNA(Cys)	0.0066	tRNA(Cys)	0.0066
Gln-tRNA(Gln)	0.1054	tRNA(Gln)	0.1054
Glu-tRNA(Glu)	0.3018	tRNA(Glu)	0.3018
Gly-tRNA(Gly)	0.2904	tRNA(Gly)	0.2904
His-tRNA(His)	0.0663	tRNA(His)	0.0663
Ile-tRNA(Ile)	0.1927	tRNA(Ile)	0.1927
Leu-tRNA(Leu)	0.2964	tRNA(Leu)	0.2964
Lys-tRNA(Lys)	0.2862	tRNA(Lys)	0.2862
Met-tRNA(Met)	0.0507	tRNA(Met)	0.0507
Phe-tRNA(Phe)	0.1339	tRNA(Phe)	0.1339
Pro-tRNA(Pro)	0.1647	tRNA(Pro)	0.1647
Ser-tRNA(Ser)	0.1854	tRNA(Ser)	0.1854
Thr-tRNA(Thr)	0.1914	tRNA(Thr)	0.1914
Trp-tRNA(Trp)	0.0284	tRNA(Trp)	0.0284
Tyr-tRNA(Tyr)	0.1020	tRNA(Tyr)	0.1020
Val-tRNA(Val)	0.2646	tRNA(Val)	0.2646
ATP	59.2760	ADP	59.2760
H${}_{2}$O	59.2760	phosphate	58.70001
(1→3)-β-D-glucan	1.1348	H${}^{+}$	59.3050
(1→6)-β-D-glucan	1.1348	biomass	1
glycogen	0.5185		
trehalose	0.0234		
mannan	0.8079		
riboflavin	0.00099		
lipid	1		
sulphate	0.0200		
dAMP	0.0036		
dCMP	0.0024		
dGMP	0.0024		
dTMP	0.0036		
AMP	0.0460		
CMP	0.0447		
GMP	0.0460		
UMP	0.0599		

**Table A4:** Helper reaction for producing the protein quota in the Yeast 6.06 model.

Reactants	Stoichiometry	Products	Stoichiometry
Ala-tRNA(Ala)	0.9839201541	tRNA(Ala)	0.9839201541
Arg-tRNA(Arg)	0.3446294001	tRNA(Arg)	0.3446294001
Asn-tRNA(Asn)	0.2181008711	tRNA(Asn)	0.2181008711
Asp-tRNA(Asp)	0.6380040232	tRNA(Asp)	0.6380040232
Cys-tRNA(Cys)	0.0141540388	tRNA(Cys)	0.0141540388
Gln-tRNA(Gln)	0.2260357111	tRNA(Gln)	0.2260357111
Glu-tRNA(Glu)	0.6472255939	tRNA(Glu)	0.6472255939
Gly-tRNA(Gly)	0.6227777087	tRNA(Gly)	0.6227777087
His-tRNA(His)	0.1421837537	tRNA(His)	0.1421837537
Ile-tRNA(Ile)	0.4132550429	tRNA(Ile)	0.4132550429
Leu-tRNA(Leu)	0.6356450167	tRNA(Leu)	0.6356450167
Lys-tRNA(Lys)	0.6137705931	tRNA(Lys)	0.6137705931
Met-tRNA(Met)	0.1087287529	tRNA(Met)	0.1087287529
Phe-tRNA(Phe)	0.2871554242	tRNA(Phe)	0.2871554242
Pro-tRNA(Pro)	0.3532076054	tRNA(Pro)	0.3532076054
Ser-tRNA(Ser)	0.3975998181	tRNA(Ser)	0.3975998181
Thr-tRNA(Thr)	0.4104671262	tRNA(Thr)	0.4104671262
Trp-tRNA(Trp)	0.060905258	tRNA(Trp)	0.060905258
Tyr-tRNA(Tyr)	0.2187442365	tRNA(Tyr)	0.2187442365
Val-tRNA(Val)	0.5674482841	tRNA(Val)	0.5674482841
ATP	36.3823134562	ADP	36.3823134562
H${}_{2}$O	36.3823134562	phosphate	36.3823134562
		H${}^{+}$	36.3823134562
		protein quota	1
