# Supplementary material for: A Protocol for Generating and Exchanging (Genome-Scale) Metabolic Resource Allocation Models
Source: Metabolites. 2017 Sep 6;7(3):47. doi: 10.3390/metabo7030047 (PMC5618332; doi:10.3390/metabo7030047)
Supplement: Supplementary file 1 [file metabolites-07-00047-s001.zip › metabolites-217957-publish-supplement/supplement.pdf]

Supplementary Material for: “A Protocol for Generating and Exchanging (Genome-Scale) Metabolic Resource Allocation Models”

Alexandra-Mirela Reimers, Henning Lindhorst, Steffen Waldherr

September 4, 2017

1 Resource allocation model

The resource allocation model used as example for Section 8 is given in Table 1.

The linear program derived from this model is described in Section 2 of the main paper. The SBML file in the ’ram’ standard can also be found in the supplements. Here we want to simply present the matrices H_C, H_E, H_B, and H_M s.t. the readers can check their implementations.

1.1 Enzyme capacity constraint

The enzyme capacity constraint is formulated as

$$H_C \cdot v(t) \leq H_E p(t)$$

Please note that we are printing the transposed matrix.

$$H_C^T = \begin{pmatrix}
\text{5.56e-04} & -\text{5.56e-04} & 0.00 & 0.00 & 0.00 & 0.00 & 0.00 & 0.00 & 0.00 \\
0.00 & 0.00 & \text{4.17e-04} & -\text{4.17e-04} & 0.00 & 0.00 & 0.00 & 0.00 & 0.00 \\
0.00 & 0.00 & 0.00 & 0.00 & 0.00 & 0.00 & \text{5.00e-04} & 0.00 & 0.00 \\
0.00 & 0.00 & 0.00 & 0.00 & 0.00 & \text{4.00e-04} & 0.00 & 0.00 & 0.00 \\
0.00 & 0.00 & 0.00 & 0.00 & 0.00 & \text{5.00e-04} & 0.00 & 0.00 & 0.00 \\
0.00 & 0.00 & 0.00 & 0.00 & 0.00 & 0.00 & 0.00 & 0.00 & 0.00 \\
0.00 & 0.00 & 0.00 & 0.00 & \text{2.00e-01} & 0.00 & 0.00 & 0.00 & 0.00 \\
0.00 & 0.00 & 0.00 & 0.00 & 0.00 & 0.00 & 0.00 & \text{4.00e-02} & -\text{3.33e-02} \\
0.00 & 0.00 & 0.00 & 0.00 & \text{1.00e-01} & 0.00 & 0.00 & 0.00 & 0.00 \\
0.00 & 0.00 & 0.00 & 0.00 & \text{5.99e-02} & 0.00 & 0.00 & 0.00 & 0.00 \\
0.00 & 0.00 & 0.00 & 0.00 & \text{1.60e-01} & 0.00 & 0.00 & 0.00 & 0.00 \\
0.00 & 0.00 & 0.00 & 0.00 & \text{1.60e-01} & 0.00 & 0.00 & 0.00 & 0.00 \\
0.00 & 0.00 & 0.00 & 0.00 & \text{2.00e-01} & 0.00 & 0.00 & 0.00 & 0.00 \\
0.00 & 0.00 & 0.00 & 0.00 & \text{1.00e+00} & 0.00 & 0.00 & 0.00 & 0.00 \\
0.00 & 0.00 & 0.00 & 0.00 & \text{1.00e-01} & 0.00 & 0.00 & 0.00 & 0.00
\end{pmatrix}$$

The negative entries derive from reversible reactions in the model.

For the construction of the filter matrix, we define the species vector as

$$p^T = (\text{Stor, Complex\_1, Emetab1, Emetab2, Estor, Etrans1, Etrans2, R, S}).$$

External metabolites:	N_1, N_2, O_2
Internal metabolites:	N, AA, ATP
Macromolecules:	Stor, ETrans1, Complex1, EMetab1, EMetab2, EStor, S, R

No.	Reactions		Catalysed by	Turnover rate
1	$N_1 + O_2$	$\leftrightarrow$ N	ETrans1	1800
2	$N_2 + O_2$	$\leftrightarrow$ N	Complex1	2400
3	N	$\rightarrow$ AA + ATP	EMetab1	2000
4	N	$\rightarrow$ AA + ATP	EMetab2	2500
5	N	$\rightarrow$ AA + 2 ATP	EMetab2	2000
6	50 AA + 60 ATP	$\rightarrow \varnothing$		
7	200 AA + 300 ATP	$\leftrightarrow$ Stor	EStor	f: 25, r: 30
8	100 AA + 400 ATP	$\rightarrow$ ETrans1	R	10
9	160 AA + 640 ATP	$\rightarrow$ Complex1	R	6.25
10	200 AA + 800 ATP	$\rightarrow$ EMetab1	R	5
11	160 AA + 640 ATP	$\rightarrow$ EMetab2	R	6.25
12	150 AA + 500 ATP	$\rightarrow$ EStor	R	5
13	1500 AA + 200 ATP	$\rightarrow$ S	R	10
14	1000 AA + 4000 ATP	$\rightarrow$ R	R	1

Table 1: List of species, reactions, and catalysis relationships for the toy model whose SBML representation can be found in the Supplement. The reaction that consumes AA and ATP but produces nothing is the maintenance reaction and is considered spontaneous. The reaction No. 7 has different turnover rates for the forward (f) and reverse (r) directions. Complex1 is a transporter complex. S is a structural quota component and R is the ribosome.

The filter matrix H_E then reads

$$H_E = \begin{pmatrix} 0 & 0 & 0 & 0 & 1 & 0 & 0 & 0 \\ 0 & 0 & 0 & 0 & 1 & 0 & 0 & 0 \\ 1 & 0 & 0 & 0 & 0 & 0 & 0 & 0 \\ 1 & 0 & 0 & 0 & 0 & 0 & 0 & 0 \\ 0 & 0 & 0 & 0 & 0 & 0 & 1 & 0 \\ 0 & 0 & 1 & 0 & 0 & 0 & 0 & 0 \\ 0 & 1 & 0 & 0 & 0 & 0 & 0 & 0 \\ 0 & 0 & 0 & 1 & 0 & 0 & 0 & 0 \\ 0 & 0 & 0 & 1 & 0 & 0 & 0 & 0 \end{pmatrix}$$

1.1.1 Biomass composition constraint

The SBML file for the resource allocation model also defines a biomass composition constraint enforcing 20% of the biomass to be dedicated to the quota component S. The

biomass composition constraint then reads

$$H_B \begin{pmatrix} c(t) \\ p(t) \end{pmatrix} \leq 0,$$

with the storage species $\mathcal{C} = \mathrm{Stor}$ and the constraint matrix

$$H_B = \begin{pmatrix} 0.03 & 0.024 & 0.03 & 0.024 & 0.0225 & 0.015 & 0.009 & 0.15 & -1.275 \end{pmatrix}.$$

1.1.2 Maintenance reaction

The reaction No. 6 is a maintenance reaction coupled to the total biomass in the system. The corresponding constraint in deFBA is given as

$$v(t) \geq H_M \begin{pmatrix} c(t) \\ p(t) \end{pmatrix}, \tag{1}$$

with the matrix H_M all zeros except for the sixth row

$$H_{M,6} = \begin{pmatrix} 0.04 & 0.032 & 0.04 & 0.032 & 0.03 & 0.02 & 0.012 & 0.2 & 0.3 \end{pmatrix}$$
